# Non-Coding RNA-Driven Regulation of rRNA Biogenesis

**DOI:** 10.3390/ijms21249738

**Published:** 2020-12-20

**Authors:** Eleni G. Kaliatsi, Nikoleta Giarimoglou, Constantinos Stathopoulos, Vassiliki Stamatopoulou

**Affiliations:** Department of Biochemistry, School of Medicine, University of Patras, 26504 Patras, Greece; e_kaliatsi@upatras.gr (E.G.K.); n.giarimoglou@upatras.gr (N.G.)

**Keywords:** rRNA biogenesis, non-coding RNAs, regulatory RNAs, nucleolus

## Abstract

Ribosomal RNA (rRNA) biogenesis takes place in the nucleolus, the most prominent condensate of the eukaryotic nucleus. The proper assembly and integrity of the nucleolus reflects the accurate synthesis and processing of rRNAs which in turn, as major components of ribosomes, ensure the uninterrupted flow of the genetic information during translation. Therefore, the abundant production of rRNAs in a precisely functional nucleolus is of outmost importance for the cell viability and requires the concerted action of essential enzymes, associated factors and epigenetic marks. The coordination and regulation of such an elaborate process depends on not only protein factors, but also on numerous regulatory non-coding RNAs (ncRNAs). Herein, we focus on RNA-mediated mechanisms that control the synthesis, processing and modification of rRNAs in mammals. We highlight the significance of regulatory ncRNAs in rRNA biogenesis and the maintenance of the nucleolar morphology, as well as their role in human diseases and as novel druggable molecular targets.

## 1. Introduction 

**Figure 1:**
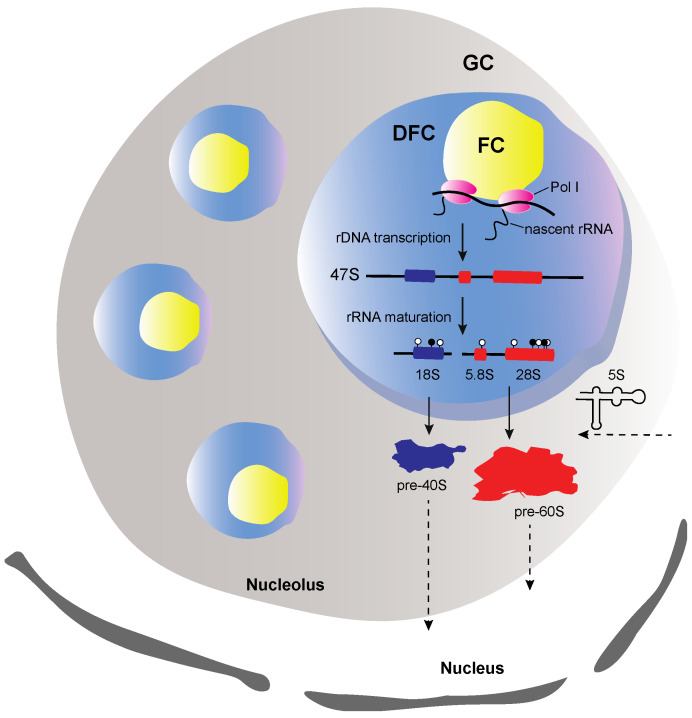
Illustration of the structure a well-defined nucleolus. The nucleolus consists of three sub-compartments, the fibrillar center (FC), the dense fibrillar center (DFC) and the granular component (GC). FC is surrounded by DFC and both are embedded in GC. At FC borderline the rDNA transcription occurs. The early and late steps of the rRNA processing occur, respectively, at DFC and GC. The early assembly steps occur in GC, where the precursors of 40S (pre-40S) and 60S (pre-60S) subunits are formed, as soon as the 5S rRNA enters the nucleolus. Pol I refers to RNA polymerase I, white and black circles refer to uridylated and methylated modified nucleotides, respectively. Arrows with dashed lines indicate transport from or to the nucleolus.

The eukaryotic ribosome as a multimolecular machine composed of more than 80 proteins and four distinct types of ribosomal RNAs (rRNAs) requires a dedicated and reliable procedure to become efficient and functional. Therefore, to achieve a consistent coordination of ribosome’s components transcription, translation and assembly, the eukaryotic cell has allocated a separate compartment, the nucleolus, where most of ribosome’s maturation steps take place [1]. The nucleolus is formed around the nucleolar organizer regions (NORs), which in human correspond to about 200 rRNA genes, localized on the five acrocentric chromosomes and represents the most distinctive cellular organelle in the interphase nucleus [2,3]. Although, being a highly dynamic condensate, the nucleolus is composed of three distinct structural entities; the fibrillar center (FC) which is surrounded by the dense fibrillar center (DFC) and both are embedded in the granular component (GC) [4]. Interestingly, these well-defined subnucleolar compartments correspond to places where successively different stages of rRNA biogenesis occur, starting from the rDNA transcription that occurs in the FC/DFC interface up to the early pre-60S and pre-40S assembly in the GC (Figure 1). It is now evident that these three compartments obey to liquid-liquid phase separation mechanisms and their formation is driven by weak RNA-protein interactions [5,6,7,8]. In particular, it has been, very recently described that prominent nucleolar proteins, like fibrillarin and nucleophosmin interact with precursor rRNA molecules (pre-rRNA) through intrinsically disordered regions and lead to pre-rRNA phase separation and consecutively to the sub-nucleolar compartments genesis [5,6,7]. However, how the nucleolus assembly and breakdown are modulated remains an open debate. 

The function and the structural organization of the nucleolus appear as two components intimately connected. Deregulation at any distinct step of ribosome biogenesis provokes the nucleolar stress response that leads to cell cycle arrest, and finally, to programmed cell death [9,10]. Hence, the nucleolar disorganization within a cell, mirrors an impaired ribosome biogenesis, which in turn has been tightly linked to various disorders [11,12]. For example, during viral infection several different viruses, including coronaviruses, recruit nucleolar proteins to favor the virus replication, causing redistribution of major nucleolar factors, including nucleolin, fibrillarin or nucleophosmin that lead to alterations of the nucleolar morphology, such as enlargement of FCs [13,14,15,16]. On the other hand, changes on the morphology and size of the nucleolus have been tightly correlated to diverse types of tumors and tracking the nucleolus phenotype has been previously reported as a reliable prognostic biomarker. It has been reported in many human tumors that the nucleolus enlargement is tightly linked to a poor clinical outcome [11,17]. Altogether, methods such as the iNo scoring tool, have been developed to evaluate both quantitatively and qualitatively the nucleolar morphology, and therefore the defective rRNA biogenesis, in an attempt to introduce phenotypic changes of the nucleolus as a predictive biomarker in disease diagnostics [18,19].

Apart from the housekeeping rRNAs, mRNAs and tRNAs, over the past few years, novel ncRNAs exhibiting non-canonical functions have drawn the attention and opened new important biological questions. RNA-mediated mechanisms have been now described in all kingdoms of life with multiple regulatory roles in chromatin remodeling, cell cycle arrest, gene expression regulation at both the transcriptional and translational level and post-transcriptional modifications, usually providing a better understanding of human diseases, including cancer [20,21,22]. Overall, growing evidence indicate that the complex landscape of the rRNA biogenesis, in combination to the nucleolar function is further regulated not only by important enzymes and scaffold proteins, but also by multiple ncRNAs [23,24,25]. Nowadays, we recognize the existence of innumerable metabolically stable RNAs, 60–300 nucleotide long, the so-called small nucleolar RNAs (snoRNAs). Although snoRNAs are produced in the nucleoplasm by RNA polymerase II, they act in the nucleolus impacting on rRNA synthesis and processing [26]. Moreover, several other sense or antisense ncRNAs, such as rDNA intergenic transcripts the synthesis of which is driven either by RNA pol I or pol II, nucleolar-specific long ncRNAs and circular RNAs, are involved in the regulation of rDNA transcription, rRNA maturation, post-transcriptional modifications and nucleolar disruption [25,27,28]. Of note, aberrant expression of most of them have been lately correlated to a plethora of ribosomopathies or diverse types of cancer and gained appreciation as potential novel modifiers that can be regulated by drugs [25]. Taken together, the emerging data point towards the multilevel regulation of ribosome biogenesis and highlight the existence of novel diagnostic and therapeutic avenues. Herein, we provide an up-to-date overview of the function of ncRNAs known to interfere with rRNA biogenesis. We focus on steps that take place inside the nucleolus, including rDNA transcription, rRNA processing and modification, and on the nucleolar disaggregation, in correlation to the development of human disorders. 

## 2. Negative Regulators of rDNA Transcription

Sense or antisense long ncRNAs (lncRNAs) derived from intergenic spacer sequences (IGS) that separate the rDNA gene clusters appear as key players in regulating directly the structural integrity of the nucleolus, as well as the rDNA transcription. 

### 2.1. pRNA 

**Figure 2:**
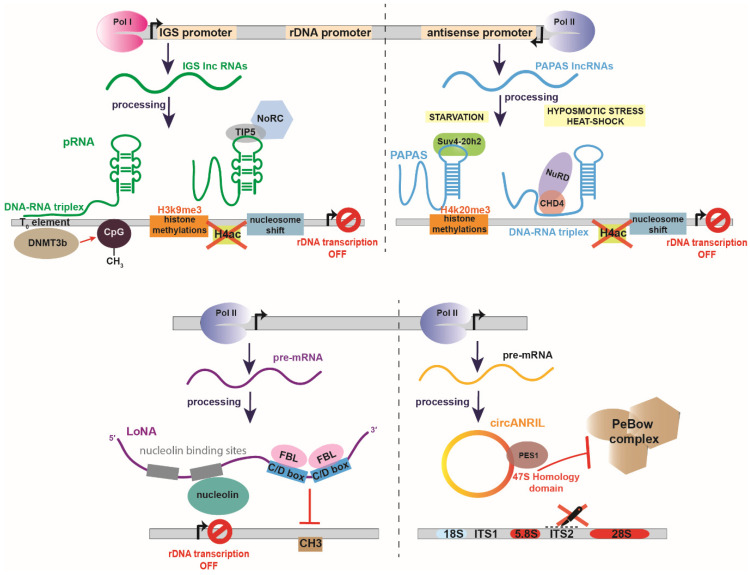
Examples of negative non-coding RNA regulators of rRNA biogenesis. (Top left panel) pRNA is transcribed by RNA polymerase I (Pol I) by an intergenic spacer promoter (IGS promoter). pRNA is a bifunctional lncRNA which 5′ end forms a DNA-RNA triplex with the T_o_ element of the rDNA promoter and the methyltransferase DNMT3b which in turn methylates CpG. At its 3′ end, pRNA forms a stem loop structure which interacts with TIP5 component of the nucleolar remodeling complex (NoRC) to induce H3K9me3 histone methylation, removal of H4ac acetylation marks and transcription inhibition by nucleosome shift. (Top right panel) Promoter and pre-antisense lncRNAs (PAPAS) are transcribed by RNA polymerase II (Pol II) from rDNA sequence in an antisense orientation. Under starvation conditions PAPAS interact with the histone methyltransferase Suv4-20h2, leading to trimethylation of histone H4 at lysine 20 (H4K20me3) and chromatin condensation. Under hypoosmotic stress or heat-shock, PAPAS interact with CHD4 and the nucleosome remodeling and deacetylase (NuRD) complex and promote histone deacetylatation, nucleosome shifts that inhibit the rDNA transcription. (Bottom left panel) Long nucleolar RNA (LoNA) is a dual-function lncRNA, transcribed by RNA pol II. LoNA contains two nucleolin-binding sequences, one of which is able to bind and suppress nucleolin’s activity. On the 3′ end, LoNA contains two C/D box sequences that compete for fibrillarin’s binding with canonical C/D box snoRNPs and suppress the pre-rRNA methylation. (Bottom right panel) CircANRIL is a circular lncRNA transcribed by RNA pol II. CircANRIL bears a homology domain with the 47S pre-rRNA which binds PES1, a component of PeBoW complex. Binding of PES1 by circANRIL suppresses PeBoW formation and inhibits ITS2 cleavage. ITS1 refers to Internal transcribed spacer 1 and ITS2 to Internal transcribed spacer 2.

The promoter-associated lncRNAs (pRNAs, 150–300 nt length) are ribosomal IGS transcripts that modulate epigenetically the rDNA transcription. pRNA is a dual-function lncRNA which production is driven by an alternative rDNA promoter located upstream of the main one, and requires RNA pol I and exosome action to be rendered functional [29,30]. The special feature of a pRNA is that forms a stem-loop structure which allows the interaction with TIP5, component of the nucleolar remodeling complex (NoRC). The recruitment of NoRC to rDNA promotes H3K9me3 histone methylations and removal of H4ac acetylation marks but also causes a nucleosome shift that blocks RNA pol I access to the rDNA promoter [31]. In addition, pRNA’s dual functionality lies in its 5′ part that forms a DNA:RNA triplex helix with the T_0_ regulatory element of the rDNA promoter which altogether redirects the DNA methyltransferase DNMT3b to methylate CpGs at the rDNA promoter (Figure 2) [29,32]. Consistent with these data, knockdown of pRNAs results in disruption of NoRC nucleolar localization, decrease of the DNA methylation level and enhancement of the rDNA transcription [29]. Notably, recent studies highlight pRNAs contribution to the initiation of differentiation by condensing the open chromatin at rDNA loci of embryonic stem cells (ESCs) and loss of their pluripotency [33]. In line with this, it has been reported that the genomic stability necessitates the presence of pRNAs to trigger the heterochromatin formation at telomeres and centromeres [34]. 

### 2.2. PAPAS

PAPAS (Promoter and pre-rRNA antisense) are another type of IGS transcripts transcribed by RNA pol II from the rDNA clusters in an antisense orientation. Mechanistically, PAPAS are expressed in response to stress conditions and by being complementary to both the rDNA promoter and rDNA coding sequences lead to inhibition of rDNA transcription and therefore, to cell survival [35].

More specifically, PAPAS, instead of recruiting Suv4-20h2 at rDNA loci, interact with complementary rDNA sequence via the formation of a DNA-RNA triplex with the dephosphorylated by heat shock ATPase CHD4, a NuRD (Nucleosome Remodeling and Deacetylase) complex subunit [36]. Once directed to rDNA, NuRD deacetylates histones and shifts the rDNA promoter-bound nucleosomes by 24 nucleotides downstream to a position that does not favor the initiation of transcription (Figure 2) [37].

In general, specific ribosomal IGS lncRNAs of about 300–400 nucleotides long, are expressed as a response to diverse environmental stimuli, resulting in transcriptionally inactive nucleoli [38]. Particularly, heat shock leads to accumulation of IGS_16_ and IGS_22_ lncRNA, while acidosis due to accumulation of IGS_28_, results in loss of nucleolar structure. Such IGS transcripts interact with the nucleolar detention signal (NoDS) (consisting of the motif RR^L/I^ and at least two hydrophobic Lh^L/V^ triplets; h refers to any hydrophobic residue) of various proteins. Thus, cell cycle regulators, E3 ubiquitin ligases and chaperons, are immobilized inside the nucleolus, causing a nucleolar reorganization (a characteristic “nucleolar caps” phenotype) and rRNA synthesis arrest [39].

### 2.3. LoNA

In contrast to the IGS transcripts, a different bifunctional nucleolar-specific lncRNA that is transcribed by RNA pol II and was recently identified in neurons, is LoNA [40,41]. LoNA’s 5′ domain harbors two nucleolin-binding sites. Nucleolin, one of the most abundant proteins in the nucleolus, contributes to multiple stages of ribosome synthesis, including rDNA transcription, pre-rRNA processing and editing, as well as to the exonuclear shuttle of ribosomal proteins and the ribosome assembly (Figure 2) [42,43,44,45]. Hence, LoNA suppresses the nucleolin multifunctional activity both in vitro and in vivo by direct binding to nucleolin. Moreover, the 3′ part of LoNA contains two C/D box sequences through which competes with U3 snoRNA for fibrillarin binding leading to decrease of 2′-O methylation of rRNAs. Conversely, decrease of LoNA results in increased rRNA synthesis and protein translation rates [40].

Deficiency in rRNA synthesis and nucleolar stress are now considered as early signs of aging and neurodegenerative disorders [41,46]. Strikingly, LoNA’s expression levels have been found elevated in Alzheimer’s disease mice, while LoNA’s decrease in their hippocampus not only improved long-term memory but also restored their learning ability. Overall, these data highlight LoNA as an important negative regulator of ribosome biogenesis that could be exploited as a novel molecular target for neurological diseases treatment and cure. 

## 3. Positive Regulators of rDNA Transcription

### 3.1. DISNOR187/238

A real proof of an RNA pol II-mediated regulation of rRNA biogenesis is found in studies on the rDNA-derived long non-coding RNAs termed disnor187 and disnor238 [47]. In the periphery of the nucleolus where the rDNA arrays are anchored, a distal flanking sequence (DJ) at the telomeric side of the acrocentric chromosomes has been proved to be transcriptionally active. DJ sequences are highly conserved among the five human acrocentric chromosomes and consist of tandem repeats of approximately 100 kb. Two promoter sequences are embedded, starting at 187 kb and 238 kb and lead to the production of disnor187 and disnor238, respectively. Their depletion in human cells stimulates the nucleolar stress response pathway and suppresses the rDNA transcription, suggesting a regulatory role on the rRNA biogenesis and genome stability maintenance; however, the exact mechanism is still elusive. Moreover, the localization of disnor187 and disnor238 in the short arms of acrocentric chromosomes, raise questions regarding their potential connection to human diseases [11,48].

### 3.2. SLERT

**Figure 3:**
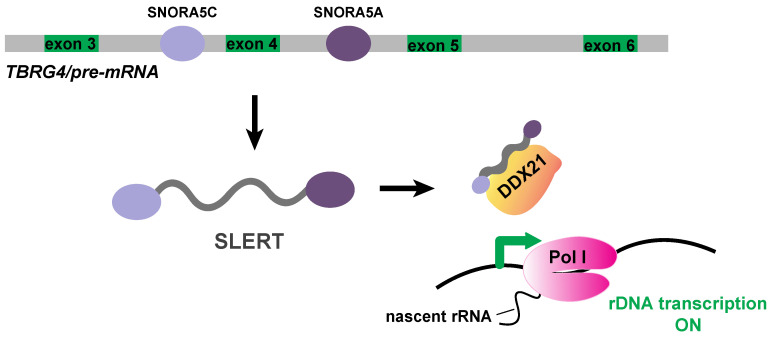
SLERT, a positive regulator of rDNA transcription. SLERT (snoRNA-ended lncRNA enhancing pre-ribosomal RNA transcription) is a snoRNA-ended lncRNA, derived from alternative splicing of TGFB4 pre-mRNA and contains two box H/ACA snoRNAs, the SNORA5C and SNORA5A sequences, at its 5′ and 3′ end, respectively. SLERT enters the nucleolus, binds to DDX21 to alter its conformation, permitting RNA pol I (Pol I) to transcribe the rDNA gene.

Among the small nucleolar RNAs involved in the regulation of rDNA transcription, a specific 694nt transcript has been strongly detected in human embryonic stem cells (hESCs), but also in various human cell lines and is termed SLERT (snoRNA-ended lncRNA enhances pre-ribosomal RNA transcription) [49]. This transcript has been characterized as a H/ACA snoRNA originating from alternative splicing of the intronic region of TBRG4 (Transforming Growth Factor Beta Regulator 4) pre-mRNA. SLERT contains the sequence of SNORA5A and SNORA5C snoRNAs at the 3′ and 5′ end, respectively, which are required for SLERT translocation into the nucleolus. SLERT promotes the rDNA transcription by binding the DDX21 RNA helicase which recruits snoRNAs involved in the early and late step of pre-40S biogenesis [50,51]. SLERT-DDX21 complex regulates rDNA transcription through an allosteric mechanism. Specifically, SLERT binds to ATP binding domain of DDX21 and by loosening DDX21 ring-shaped conformation that normally surrounds RNA pol I, allows the initiation of the rDNA transcription [49] (Figure 3). SLERT’s potential role in tumor growth suppression is also noteworthy, since its depletion can lead to remarkable reduction of the proliferation rate of the cancer cells. 

### 3.3. AluRNA

**Figure 4:**
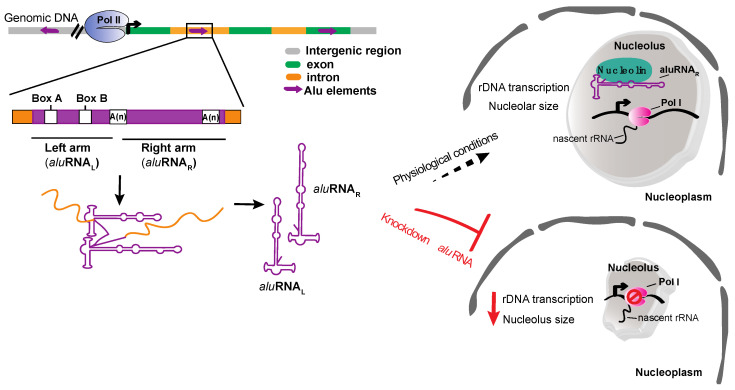
aluRNAs role in promoting the rDNA transcription. *alu*RNAs are transcribed from *Alu* elements located in intronic regions by RNA pol II (Pol II). *alu*RNAs are found as separated monomer units (*alu*RNA_L_ and *alu*RNA_R_) in cells and could be accumulated in the nucleolus. Under physiological conditions, *alu*RNAs bind to nucleolin and promote the rDNA transcription. On the other hand, knockdown of *alu*RNAs causes disruption of the nucleolar structure and reduces the rDNA transcription.

*Alu* elements were first identified as transposable elements, with specific role on the regulation of gene expression. They are transcribed by RNA pol III and derived from the dimerization of 7SL RNA genes separated by A-tails [52,53]. Interestingly, intronic regions containing *Alu* elements generate *alu*RNAs synthesized by RNA pol II which act as positive regulators of rRNA synthesis and nucleolar structure maintenance [54,55]. The nascent transcripts form a dimeric structure which is subdivided to the left and right arm *alu*RNAs (Figure 4). However, in human transcriptome, *alu*RNAs are also found as separate monomer units, the *alu*RNA_L_ and *alu*RNA_R_, lacking the flanking sequences. Although the transcripts are produced outside the nucleolus, it has been found that these intron-encoded *alu*RNAs can be transferred into nucleoli, with *alu*RNA_R_ being the most abundantly detected. Inside the nucleolus *alu*RNAs interact with proteins like nucleolin and nucleophosmin and enhance the pre-rRNA synthesis. Indeed, knockdown of *alu*RNAs in HeLa, as well as in mouse cells, causes downregulation of the rDNA transcription and severe disruption of the nucleolus size (Figure 4) [55]. 

### 3.4. 5S-OT

Although 5S rRNA is transcribed by RNA pol III outside the nucleolus, as an essential component of ribosome, its synthesis and transport to the nucleolus is strictly regulated. Recently, it was found that the *cis*-acting lncRNA 5S-OT (5S Overlapping Transcript) can act as a connector of RNA pol II and RNA pol III transcription [56]. Hence, 5S-OT is generated from 5S rDNA locus by RNA pol II as a sense transcript that harbors a sequence complementary to 5S rRNA and stimulates the 5S rRNA transcription. However, recent studies in humans revealed that this lncRNA can also act in trans. More specifically, an altered 5S-OT lncRNA, transcribed by the 5S-OT gene where an *Alu* sequence has been inserted at its 3′ end, can bind to the splicing factor U2AF65 and modulate the alternative splicing of multiple pre-mRNAs [56]. It is intriguing that knockdown of 5S-OT suppresses human macrophage differentiation, indicating 5S-OT potential implication in human immune system development.

### 3.5. risiRNA

**Figure 5:**
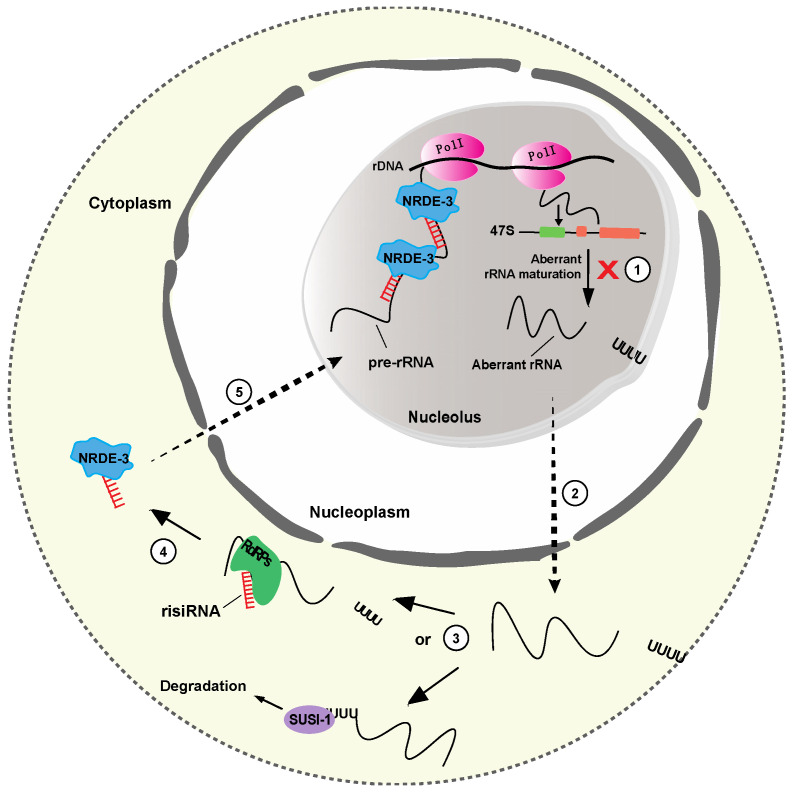
Biogenesis and function of risiRNAs in *C. elegans*. (**1**) In response to cellular stress or deficient rRNA modification processing, aberrant rRNAs accumulate and are uridylated at their 3′ end. (**2**) The uridylated rRNAs are, then, transferred to the cytoplasm, where they are either degraded by 3′-5′ exoribonuclease SUSI-1 (**3**), or, transcribed by RNA dependent RNA polymerases (RdRPs) to produce risiRNAs (antisense ribosomal siRNAs) (**4**). risiRNAs can then interact with Argonaute proteins, like NRDE, and are transported to the nucleolus, where they silence the rDNA transcription (**5**).

The discovery of ribosomal siRNAs (risiRNAs) unveiled an additional layer of gene expression regulation that involves direct cooperation of rRNA biogenesis and the RNAi pathway. The role of risiRNAs has been extensively studied in *Caenorhabditis elegans*, where these RNAs are produced under specific stress conditions. Upon cold shock, UV exposure or deficient rRNA processing, aberrant nascent rRNA is marked by oligouridylation and transferred to the cytoplasm. There, the oligouridylated rRNA can follow two different pathways; it can be either surveilled and degraded by specific 3′-5′ exonucleases, like *SUSI-1 in C. elegans or* DIS3L in mammals [57,58] or can act as a template for RNA-dependent RNA polymerases (RdRPs) to generate risiRNAs (Figure 5). In *C. elegans*, risiRNAs bear a 22nt long sequence complementary to 18S and 26S rRNAs, through interaction with Argonaute (AGO) proteins, like NRDE. Upon complex formation, they are transported to the nucleolus and bind the nascent rRNA to further silence the rDNA transcription (Figure 5) [59,60]. Despite risiRNAs protective role in the accumulation of aberrant rRNAs, their exact biogenesis pathway along with their biological significance or potential involvement to human diseases, remain currently essentially unexplored. 

## 4. Negative Regulators of rRNA Processing

### circANRIL

Circular RNAs (CircRNAs) are a recently described and broadly studied class of ncRNAs. CircRNAs were first identified in the late ‘70s, but it was not until 2013 when they were considered as a new family of regulatory RNA molecules. They are single-stranded RNAs with covalently closed circular isoforms and can function as microRNA (miRNA) sponges [61,62,63], protein scaffolds [64,65] and templates for protein translation [66,67,68,69] in various pathophysiological processes. In a recent report, circANRIL (circular antisense long non-coding RNA in the INK4 locus) was found to regulate the maturation of pre-rRNA through binding to the nucleolar protein pescadillo 1 (PES1) [70] (Figure 2). PES1 is a member of the PES1-BOP1-WDR12 (PeBoW) complex, which is essential for 47S pre-rRNA processing and cleavage of internal transcribed spacer 2 (ITS2) [71]. Therefore, PES1 by binding to circANRIL at a 47S pre-rRNA homology domain blocks the PeBoW complex formation leading to nucleolar stress. Overexpression of circANRIL in HEK-293 cells leads to a severe nucleolar stress, indicated by an increased number of smaller nucleoli along with an accumulation of 36S and 32S pre-rRNA intermediates and a decreased proliferation rate and apoptosis [70]. Taken together, circANRIL, in contrast to linear ncRNAs, is particularly stable against degradation and has been suggested as a potential and promising atheroprotective agent, since its apoptotic effect was especially enhanced in human vascular cells and tissues. The clinical value of circANRIL is also supported by its low abundance in children suffering from Kawasaki disease [72].

## 5. Positive Regulators of rRNA Processing

### 5.1. snoRNAs

**Figure 6:**
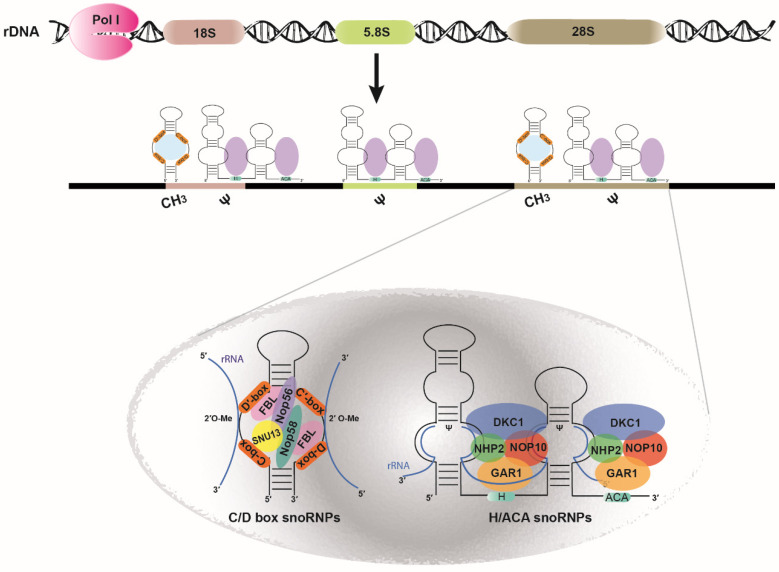
Schematic representation of snoRNAs involved in pre-rRNA modification. C/D and H/ACA snoRNPs (small nucleolar ribonucleoproteins) hybridize on pre-rRNA at specific sites that undergo 2′−O methylation and pseudouridylation, respectively. *(Lower panel)* The C/D box snoRNA contains the conserved C and D motifs where the site-specific 2′-O methylation of the target rRNA is catalyzed by fibrillarin (FBL). In addition, for an active C/D snoRNP, the proteins SNU13 (15.5K), NOP58 and NOP56 are also required. The H/ACA snoRNAs harbor the conserved H and ACA elements that form two pseudouridylation pockets, where pseudouridylation of the target rRNA is catalyzed by dyskerin (DKC1). The core proteins NHP2, NOP10, GAR1 are essential for a functional H/ACA snoRNP.

The complex landscape of rRNA biogenesis is further enriched by several epigenetic and post-transcriptional events, mediated by small nucleolar RNAs (snoRNAs). SnoRNAs are 60–300 nucleotides in size and guide the maturation and post-transcriptional modification of rRNAs [73,74,75,76]. Intriguingly, in vertebrates most snoRNAs rather than being transcribed from independent genes are processed from introns of protein-coding or non-coding genes. The transcription of a few snoRNAs occurs autonomously by RNA pol II [74]. Briefly, snoRNAs are co-transcribed with the host gene and processed by splicing and exonucleolytic digestion. The nascent intronic snoRNAs are then being assembled with ribonucleoproteins that appear essential for both processing stability and nucleolar localization of snoRNPs (small nucleolar ribonucleoproteins) [74,77]. SnoRNAs are divided into two major classes with distinctive, evolutionarily conserved sequence elements, including the C/D box snoRNAs that guide the 2′-O ribose methylation to stabilize rRNA helices and H/ACA box snoRNAs that direct the isomerization of uridine to pseudouridine and permit additional hydrogen bonding, leading to a more rigid ribosome structure (Figure 6) [73]. In the yeast *Saccharomyces cerevisiae* 45 ψ and 55 2′-O methylation sites have been identified on rRNAs and around 100 of each are found in human rRNAs [78,79,80,81]. 

More specifically, at the 5′ and 3′ ends of the box C/D family two conserved sequence elements exist: the box C (RUGAUGA) and D (CUGA) motifs, respectively. Τhis family is characterized by a kink-turn (k-turn) structure [82], which is necessary for the formation of the C/D box snoRNP. A C/D box snoRNP consists of Fibrillarin (FBL) which bears the catalytic methyltransferase activity and the protein factors SNU13(15.5K), NOP58 and NOP56 [74,83,84]. Among the family of C/D box snoRNAs, U8, a vertebrate specific snoRNA, is involved in 5.8S and 28S rRNAs maturation, whereas U3, U13, U14 and U22 are necessary for 18S rRNA processing. The U3 snoRNP is the most well characterized and is essential for the assembly of the small subunit processome, an essential complex for 18S rRNA and 40S subunit’s maturation. The interaction between the 5′ end of the U3 snoRNA and 5′ ETS (External transcribed spacer) of the yeast 18S rRNA [85] is crucial for activity but the snoRNP assembly is essential for cleavage [86]. Of note, now it is considered that there is a direct link between the complete loss of specific snoRNAs or less active catalytic components of snoRNPs and the emergence of various diseases. Abrogation of a single snoRNA that alters the modification pattern of rRNAs rarely has dramatic effects, however, the loss of a snoRNA that directs the rRNA processing, is generally fatal [87,88,89]. Strikingly, the type of the resulting disease reflects the cause that leads to rRNA 2′-O methylation deficiency. Therefore, defects in the rRNA 2′-O methylation pattern caused by aberrant snoRNAs are related to carcinogenesis [90], while mutations in *TCOF1* gene, a regulator of RNA pol I, are associated with the Treacher Collins syndrome [91]. In addition, in mammals there is a bidirectional relation between fibrillarin and p53. The latter acts as a negative regulator of fibrillarin, while p53 is upregulated upon fibrillarin depletion, through triggering both the nucleolar stress response pathway and the cap-independent translation of the p53 mRNA [92,93,94,95]. Moreover, U50 snoRNA which methylates C2848 and G2863 of 28S rRNA, downregulates KRAS, playing a direct role in tumorigenesis. Interestingly, U50 snoRNA misregulation has been detected in prostate and breast cancer, and along with oncogenic *KRAS* mutations in various other types of cancer, including cutaneous melanoma, breast and lung adenocarcinomas [96,97,98].

Furthermore, H/ACA box snoRNAs adopt the “hairpin–hinge–hairpin–tail” secondary structure with the conserved box H “ANANNA” sequence (N represents any nucleotide) within the hinge region and the ACA box on the 3′ end of the last hairpin. The hairpins of H/ACA box snoRNAs are interrupted by an internal loop which possesses on each strand a short complementary sequence to the RNA substrate and create the pseudouridylation pocket [26,74,83,99]. The assembly of a functional H/ACA snoRNP includes the core proteins NHP2, NOP10, GAR1 and the dyskerin pseudouridine synthase 1 (DKC1) [100]. snR10, snR30/U17 and E3 H/ACA snoRNAs contribute to the 18S rRNA synthesis, their exact role though is yet to be elucidated. 

Moreover, deficiency of the catalytic snoRNP proteins is the cause of many diseases. For instance, mutations in DKC1 have been observed in X-linked Dyskeratosis congenita (X-DC), a disorder characterized by hematopoietic defects and cutaneous abnormalities [101]. These mutations affect the level of rRNA pseudouridylation and selectively promote the IRES-dependent translation of specific mRNAs, like that of the growth factor VEGF, which in turn explains the increased cancer risk of X-DC patients [102,103,104]. In addition, loss of SNORA24, which directs Ψ609 and Ψ863 formation on the 18S rRNA, combined with the oncogenic *RAS* expression promotes cancer [105]. Precisely, Ψ609 is located in the ribosome’s decoding center, and therefore lack of this modification leads to faulty tRNA selection and ribosome translocation. Overall, several snoRNAs with aberrant expression have been related to cancer but their exact consequences on ribosome biogenesis remain unknown [98].

A distinct group of snoRNA with no characteristic primary sequence motif forms the RNA component of RNase MRP (RMRP). In human, RMRP is transcribed by RNA pol III, while its yeast homolog, NME1 (nuclear mitochondrial endonuclease 1) is transcribed by RNA pol II [106,107]. In fact, RMRP is the catalytic subunit of the bifunctional ribonucleoprotein RNase MRP which is involved not only in the mitochondrial DNA replication, but also in the pre-rRNA cleavage at the A3 site in the nucleolus [108]. RMRP is well-conserved among organisms, but its exact role was controversial. This RNA was recently found to mediate the human pre-rRNA ITS1 (Internal transcribed spacer 1) cleavage at site 2 [109]. Approximately 100 distinct mutations in RMRP that disrupt rRNA processing have been related to cartilage-hair hypoplasia (CHH) development, a form of dwarfism [109,110,111].

### 5.2. SAMMSON

**Figure 7:**
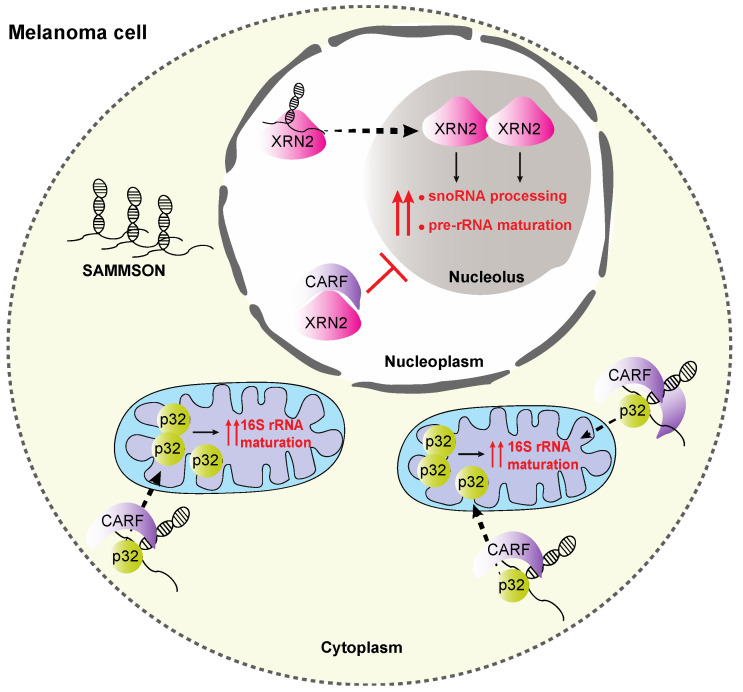
Schematic representation of SAMMSON regulatory role in pre-rRNA processing. SAMMSON is a lncRNA that is aberrantly expressed in melanoma cells. In the cytoplasm of a melanoma cell, SAMMSON interacts with CARF and then with p32, which is transferred in the mitochondria and promotes the mitochondrial 16S rRNA maturation. Moreover, in the nucleus of a melanoma cell, SAMMSON interacts with XRN2 to induce the snoRNA and pre-rRNA in the nucleolus.

Ribosome biogenesis is increased in cancer cells and coordination between the cytosolic and mitochondrial translational machineries is required to achieve a high proliferative cell rate [112]. SAMMSON, a lncRNA found aberrantly expressed in cutaneous melanoma, is considered a positive regulator of ribosome synthesis and translation, facilitating an unlimited growth rate of immortalized cells irrespectively of their tissue [113,114]. SAMMSON’s uniqueness lies in its direct involvement in both nucleolar and mitochondrial production of ribosomes. SAMMSON triggers rRNA transcription in both compartments via direct binding to CARF, XRN2 and p32 proteins (Figure 7). CARF under normal conditions binds XRN2, a 5′-3′ exoribonuclease, and prevents its transport to the nucleolus, blocking the snoRNA ends processing and the decay of the excised pre-rRNA spacer fragments [115,116,117,118]. In melanoma cells, SAMMSON allows XRN2 shuttle into the nucleolus and promotes the pre-rRNA maturation. On the other hand, it forms a complex with CARF and p32, triggering p32 localization to the mitochondria which is required for the mitochondrial 16S rRNA maturation (Figure 7) [119,120]. Altogether, SAMMSON promotes the rRNA transcription in both compartments, while its depletion leads to aberrant pre-rRNA processing intermediates. From a clinical point of view, SAMMSON expression has only been reported in cancer cells. Interestingly, SAMMSON repression results in mitochondrial protein synthesis deficiencies, and drives melanoma cells rapidly to apoptosis, independently of *BRAF*, *NRAS* or *TP53* mutational status [113].

### 5.3. rRNA-Derived Fragments (rRFs)

Recently, bioinformatics tools have provided information about a different, tRF-like (tRNA-derived fragment-like) group of ncRNAs derived from ribosomal RNA, termed rRNA-derived fragments (rRFs). The biological significance and the exact role of this novel class of ncRNAs are poorly understood and require further investigation. However, some current insights point to rRFs as possible gene expression regulators, similarly to miRNAs, or as contributors in genome stability [121]. As yet, there is no evidence of rRFs contribution to the nucleolar function or to rRNA biogenesis in eukaryotic cells. However, in *E. coli*, a fragment of domain I of 23S rRNA (GG295–343CC, 53 nt) was identified, and despite its short length, harbors two distinct non overlapping binding sites for the ribosomal proteins L4 and L24, suggesting its essential role on the rRNA folding during the early assembly of the bacterial 50S subunit [122,123,124,125]. 

## 6. Conclusions

Nucleolus integrity and rRNA biogenesis in higher eukaryotes, including man, appear far more complicated and diverse than previously thought and especially as compared to lower eukaryotes, like yeast. It is now evident that a large number of molecular species are involved and a plethora of others might participate. Among those, several important new non-coding RNAs play key roles in the rRNA biogenesis and the nucleolar assembly, including diverse rDNA intergenic spacers, snoRNAs, circular RNAs, other long non-coding RNAs or even microRNAs. Most importantly, there is now accumulating data that directly correlate the aberrant expression of several ncRNAs with the emergence of atherosclerosis, Alzheimer’s, Kawasaki disease, as well as several ribosomopathies. However, the lack of conservation and the reduced sequence similarity between closely related species makes precise RNA identification a difficult task and necessitates a focused effort to stimulate the discovery of novel human ncRNA modulators of rRNA biogenesis [39,126]. On this basis, bioinformatics analysis using available databases such as LncATLAS [127] and snoDB [128] could facilitate the identification of ncRNAs localized in the nucleolus or ncRNAs that exhibit homology with pre-rRNA species and could potentially regulate rRNA synthesis and processing through a competition mechanism, similarly to circANRIL. On the other hand, the variety of ncRNAs with key roles in diverse steps of rRNA production and the nucleolar morphology, offers the opportunity to define multiple and novel molecular targets in order to design alternative and precise therapeutic strategies. Moreover, previous protocols developed to investigate the implication of protein factors to the nucleolar destructuration, could be further exploited and adapted in order to test and elucidate the role of new ncRNAs on the nucleolar structure [19]. Overall, the increasing stream of oncologists that consider the ribosome biogenesis the “Achilles heel” of tumorigenesis and the nucleolar stress as new anticancer strategy, clearly depicts the clinical value of such ncRNAs and the imperative need for further and thorough investigation.

## Abbreviations

CHH	Cartilage-hair hypoplasia
circRNAs	Circular RNAs
circANRIL	Circular antisense long non-coding RNA in the INK4 locus
DFC	Dense fibrillar center
DJ	Distal flanking sequence
ETS	External transcribed spacer
FC	Fibrillar center
GC	Granular component
IGS	Intergenic spacer sequences
ITS	Internal transcribed spacer
lncRNAs	Long non-coding RNAs
LoNA	Long nucleolus-specific lncRNA
ncRNAs	Non-coding RNAs
NoDS	Nucleolar detention signal
NoRC	Nucleolar remodeling complex
NORs	Nucleolar organizer regions
NuRD	Nucleosome Remodeling and Deacetylase
PAPAS	Promoter and pre-rRNA antisense
pre-rRNA	Precursor ribosomal RNA
pRNAs	Promoter-associated lncRNAs
rDNA	Ribosomal DNA
RdRPs	RNA-dependent RNA polymerases
risiRNAs	Ribosomal siRNAs
RMRP	RNA component of RNAse MRP
rRFs	rRNA-derived fragments
rRNA	Ribosomal RNA
SLERT	snoRNA-ended lncRNA enhances pre-ribosomal RNA transcription
snoRNAs	Small nucleolar RNAs
snRNAs	Small nuclear RNAs
X-DC	X-linked dyskeratosis congenita
5S-OT	5S overlapping transcript
